# Expression of gastrin-releasing peptide by excitatory interneurons in the mouse superficial dorsal horn

**DOI:** 10.1186/1744-8069-10-79

**Published:** 2014-12-11

**Authors:** Maria Gutierrez-Mecinas, Masahiko Watanabe, Andrew J Todd

**Affiliations:** Institute of Neuroscience and Psychology, College of Medical, Veterinary and Life Sciences, University of Glasgow, Glasgow, G12 8QQ UK; Department of Anatomy, Hokkaido University School of Medicine, Sapporo, 060-8638 Japan

## Abstract

**Background:**

Gastrin-releasing peptide (GRP) and its receptor have been shown to play an important role in the sensation of itch. However, although GRP immunoreactivity has been detected in the spinal dorsal horn, there is debate about whether this originates from primary afferents or local excitatory interneurons. We therefore examined the relation of GRP immunoreactivity to that seen with antibodies that label primary afferent or excitatory interneuron terminals. We tested the specificity of the GRP antibody by preincubating with peptides with which it could potentially cross-react. We also examined tissue from a mouse line in which enhanced green fluorescent protein (EGFP) is expressed under control of the GRP promoter.

**Results:**

GRP immunoreactivity was seen in both primary afferent and non-primary glutamatergic axon terminals in the superficial dorsal horn. However, immunostaining was blocked by pre-incubation of the antibody with substance P, which is present at high levels in many nociceptive primary afferents. EGFP^+^ cells in the GRP-EGFP mouse did not express Pax2, and their axons contained the vesicular glutamate transporter 2 (VGLUT2), indicating that they are excitatory interneurons. In most cases, their axons were also GRP-immunoreactive. Multiple-labelling immunocytochemical studies indicated that these cells did not express either of the preprotachykinin peptides, and that they generally lacked protein kinase Cγ, which is expressed by a subset of the excitatory interneurons in this region.

**Conclusions:**

These results show that GRP is expressed by a distinct population of excitatory interneurons in laminae I-II that are likely to be involved in the itch pathway. They also suggest that the GRP immunoreactivity seen in primary afferents in previous studies may have resulted from cross-reaction of the GRP antibody with substance P or the closely related peptide neurokinin A.

## Background

Itch is an aversive sensation, distinct from pain, which is associated with the desire to scratch. Chronic itch is a common and distressing condition associated with many diseases and certain drug treatments [1], and often occurs in the absence of obvious disease [2]. Since it is difficult to treat, chronic itch represents a major unmet clinical need, and there has therefore been considerable interest in the peripheral and central mechanisms that underlie the perception of itch [3, 4].

A major advance in our understanding came with the finding that gastrin-releasing peptide (GRP, a member of the bombesin family) and its receptor, gastrin-releasing peptide receptor (GRPR) play an important role in itch [5, 6]. Specifically, it was shown that itch (but not pain) behaviour was dramatically reduced in mice lacking GRPR [5], and also in mice in which GRPR-expressing neurons in the spinal cord had been ablated by intrathecal injection of bombesin conjugated to saporin [6]. It was also reported that GRP was present in a subset of dorsal root ganglion (DRG) cells, identified by the presence of calcitonin gene-related peptide (CGRP) [5], which is thought to be expressed by all peptidergic primary afferents [7]. However, while it is generally accepted that GRP is an important mediator if itch [3], there has been debate about whether it is expressed by primary afferents. For example, several papers have reported that the level of GRP mRNA is very low in the DRG [8–10], and it has been suggested that the staining seen with GRP antibodies in primary afferents represented cross-reaction with one of two other peptides that are present in these afferents: neuromedin B (NMB) or substance P [8, 9].

There is also disagreement over whether dorsal horn neurons express GRP. Although Sun and Chen [5] found no GRP mRNA in the superficial dorsal horn of adult mice, other published studies [8, 10–15] and the Allen Brain atlas [16] have reported numerous GRP mRNA-positive cells in laminae I-II. These were found to be absent in mice lacking the transcription factors Lmx1b, Tlx3 or Gsx1/2 [11, 12, 15], which are required for normal development of the glutamatergic phenotype, suggesting that they were excitatory interneurons [17–19]. In addition, many cells containing enhanced green fluorescent protein (EGFP) are seen in this region in a BAC transgenic mouse line from the GENSAT project, Tg(GRP-EGFP), in which expression of EGFP is under control of the GRP promoter [10]. However, Liu et al. [20] have recently questioned whether the GRP mRNA in superficial dorsal horn neurons is translated into GRP.

Most (if not all) peptide-expressing primary afferents contain CGRP [7], which is restricted to primary afferents in the dorsal horn, and peptidergic primary afferent terminals can therefore be identified by the presence of CGRP [21, 22]. The axons of glutamatergic interneurons can be detected with antibodies against the vesicular glutamate transporter 2 (VGLUT2), which is thought to be expressed by all excitatory interneurons in this region [23, 24]. Despite the controversy over the source of GRP-immunoreactive axons in the superficial laminae, there have apparently been no studies to date in which co-expression of GRP with either of these markers has been investigated within the dorsal horn.

The main aim of this study was to determine whether GRP-immunoreactivity could be detected in peptidergic (CGRP^+^) afferent terminals and/or in VGLUT2^+^/CGRP^−^ boutons, which are likely to originate from local excitatory interneurons. Because many of the latter express somatostatin [23–25] we looked for co-localisation of GRP and somatostatin in VGLUT2-containing profiles. We also tested the specificity of the GRP antibody by pre-absorption with NMB and substance P. Finally, we examined tissue from the GRP-EGFP mouse line, to look for evidence that GRP was present in EGFP expressing cells, and to provide an initial characterisation of these cells.

## Methods

All animal experiments were approved by the Ethical Review Process Applications Panel of the University of Glasgow and were performed in accordance with the European Community directive 86/609/EC and the UK Animals (Scientific Procedures) Act 1986.

### Animals and general features of tissue processing

Three adult C57Bl/6 mice of either sex (21–24 g) and 2 Tg(GRP-EGFP) mice of either sex (21 g, 27 g) (GENSAT) [10] were deeply anaesthetised with pentobarbitone (30 mg i.p.) and perfused through the left ventricle with fixative that contained 4% freshly depolymerised formaldehyde in 0.1 M PB. Lumbar spinal cord segments (L2-5) were dissected out and stored for 1–5 hours in the same fixative. They were then cut into transverse or sagittal sections with a vibrating-blade microtome and immersed in 50% ethanol for 30 mins to enhance antibody penetration.

Multiple-labelling immunofluorescence staining was performed as described previously [26–28]. Briefly, sections were incubated for 1–3 days in mixtures of primary antibodies and then for 4–18 hours in species-specific secondary antibodies that were raised in donkey and conjugated to Rhodamine Red, Alexa 488, Alexa 647, Pacific Blue or biotin (Jackson Immunoresearch, West Grove, PA, USA). All secondary antibodies were diluted 1:500, apart from those conjugated to Pacific Blue or Rhodamine Red, which were diluted 1:200 and 1:100, respectively. The biotinylated antibody was revealed with Pacific Blue conjugated to avidin (1:1000; Life Technologies, Paisley, UK). All antibodies were diluted in PBS that contained 0.3% Triton X-100 and incubations were at 4°C. Following the immunocytochemical reaction, sections were mounted in anti-fade medium and stored at -20°C. Details of all primary antibodies used, their sources and concentrations are given in Table 1.

**Table 1:** **Primary antibodies used in this study**

Antibody	Species	Catalogue no	Dilution	Source
GRP	Rabbit	BML-GA1166 lot # X09015	1:500	Enzo
CGRP	Goat	BML-CA1134	1:10,000	Enzo
VGLUT2	Rabbit	135 402	1:5,000	Synaptic Systems
VGLUT2	Guinea pig	ab2251	1:5,000	Millipore
VGLUT2	Goat		1:500	M Watanabe
Somatostatin	Guinea pig		1:1,000	P Ciofi
Substance P	Rat	OBT06435	1:200	Oxford Biotech
PPTB	Guinea pig		1:1,500	P Ciofi
EGFP	Chicken	ab13970	1:1,000	Abcam
Pax2	Rabbit	716000	1:1,000	Life Technologies
PKCγ	Guinea pig		1:500	M Watanabe
NeuN	Mouse	MAB377	1:500-1000	Millipore

Sections were scanned with a Zeiss LSM 710 confocal microscope equipped with Argon multiline, 405 nm diode, 561 nm solid-state and 633 nm HeNe lasers. Scans were obtained through either a 63× or 40× oil-immersion lens (numerical aperture 1.4 and 1.3, respectively), with the aperture set to 1 Airy unit.

### Co-localisation of GRP-immunoreactivity with other axonal markers

Sections from the 3 wild-type mice were reacted with the following combinations of primary antibodies: (1) rabbit anti-GRP, goat anti-CGRP and guinea pig anti-VGLUT2, or (2) rabbit anti-GRP, guinea pig anti-somatostatin and goat anti-VGLUT2, and in each case these were revealed with different fluorochromes. For each antibody combination, 3 sections were selected from each mouse before the immunostaining was viewed at high magnification. These were then scanned through the 63× oil-immersion lens. z-stacks consisting of around 35 optical sections (z-spacing 0.3 μm) were obtained from the middle part of the dorsal horn to include the whole dorsoventral extent of laminae I-II.

The confocal image stacks from sections reacted to reveal CGRP and VGLUT2 were analysed with Neurolucida for Confocal software (MBF Bioscience, Williston, VT, USA). The outline of the grey matter was drawn, and the ventral border of the GRP plexus (which corresponded approximately to the boundary between the inner and outer parts of lamina II) was determined from a maximum intensity projection and plotted onto the drawing. A 4 × 4 μm grid was then superimposed on the image stack. A section near the middle of the z-series was initially viewed with only the VGLUT2 and CGRP channels visible, and 100 boutons that were VGLUT2-immunoreactive but lacked CGRP (VGLUT2^+^/CGRP^−^) were selected from within the GRP plexus by choosing the bouton nearest the bottom right corner of successive grid squares, starting from a square at the dorsal surface of the grey matter and progressing through the squares in a dorsal-to-ventral, and then a left-to-right, direction until the 100 boutons had been acquired [29]. The GRP channel was then viewed and the presence or absence of GRP-immunoreactivity was recorded for each of the selected VGLUT2^+^/CGRP^−^ boutons. To compensate for bias towards larger profiles in the selection process [30], the z-axis length of each selected bouton was estimated by determining the number of z-sections on which it was present [29].

In order to estimate the proportion of peptidergic afferents that were labelled with the GRP antibody, we then re-examined the sections and selected 100 CGRP^+^ boutons in the same way. These were then assessed for the presence of GRP-immunoreactivity.

Since many glutamatergic interneurons in the superficial dorsal horn express the neuropeptide somatostatin [23–25], we looked for evidence of co-expression of GRP and somatostatin in VGLUT2-immunoreactive boutons. The level of VGLUT2 in peptidergic primary afferent boutons is far lower than that seen in the axons of neuropeptide-containing excitatory interneurons [23], and we therefore initially viewed the channels representing GRP and VGLUT2, and selected 50 boutons from each section that were GRP-immunoreactive and had relatively high levels of staining for VGLUT2 (axons of presumed GRP^+^ local neurons). We then assessed these boutons for the presence of somatostatin.

### Specificity of GRP immunostaining

Since GRP shows considerable sequence similarity to NMB, and it has been suggested that GRP-immunoreactivity in the dorsal horn may in part result from cross-reactivity with NMB [8], we tested whether the immunostaining could be blocked with either GRP or NMB peptides (Phoenix Pharmaceuticals, Burlingame, CA, USA). These were added to anti-GRP antibody at its working concentration (1:500) and the resulting solutions were stored for 18 hours at 4°C before the immunoreaction was started. For each peptide, we tested 3 different concentrations: 10^−5^, 10^−6^ and 10^-7^ M. Sections were then incubated in primary and fluorescent secondary antibody, as described above.

In preliminary experiments, we had observed that if a monoclonal antibody against substance P was included in the primary antibody mixture, staining intensity with the GRP antibody was consistently reduced (data not shown). This raised the possibility that the GRP antibody could cross-react with substance P (which is expressed by many nociceptive primary afferents [31, 32]), and that this cross-reaction was reduced in the presence of substance P antibody, which would bind to substance P and thus mask antigenic sites. We therefore also tested the effect of pre-incubating the anti-GRP antibody with substance P at 10^−5^, 10^−6^ and 10^-7^ M, as described above.

### Characterisation of EGFP cells in the GRP-EGFP mouse

We initially tested whether any GRP-EGFP cells were inhibitory interneurons by looking for co-localisation with the transcription factor Pax2, which is expressed by GABAergic neurons in this region [17], and can readily be detected in adult spinal cord sections [33, 34]. Transverse sections from L4 were reacted with chicken anti-EGFP, rabbit anti-Pax2 and mouse monoclonal antibody NeuN. Three sections from each mouse were scanned through the 40× objective to produce z-stacks (20 optical sections at 1 μm z-separation). The EGFP and NeuN channels were initially viewed with Neurolucida. A threshold was applied to allow identification of GRP-EGFP cells, and the positions of these were marked on an outline of the dorsal horn. The Pax2 channel was then switched on, and the selected GRP-EGFP cells were assessed for the presence or absence of Pax2.

Since we found that some EGFP-labelled profiles resembled axonal boutons, we tested these for expression of VGLUT2 and the vesicular GABA transporter (VGAT), which are present in the boutons of excitatory and inhibitory interneurons, respectively [24]. We also determined the proportion of EGFP^+^ glutamatergic (VGLUT2^+^) boutons that were GRP-immunoreactive, and the proportion of GRP-immunoreactive non-primary glutamatergic (GRP^+^/VGLUT2^+^) boutons that contained detectable EGFP. Transverse sections from L4 were reacted with chicken anti-EGFP, rabbit anti-GRP, guinea pig anti-VGLUT2 and goat anti-VGAT, and these were revealed with different fluorochromes as described above. Three sections from each animal were scanned through the 63× lens to produce z-stacks (30–35 optical sections at 0.3 μm z-spacing) that included the central part of laminae I-IIo. We initially looked for possible coexistence of either GRP or EGFP with VGAT by examining the entire z-series scanned from each section. We then quantified the relationship between EGFP and GRP in VGLUT2^+^ boutons. The channels corresponding to VGLUT2 and EGFP were initially viewed with Neurolucida, and 50 double-labelled boutons were identified in each section. The channel corresponding to GRP was then examined, and the presence or absence of GRP-immunoreactivity in each of the selected boutons was recorded. The sections were then re-analysed: 50 GRP^+^ boutons with strong VGLUT2 immunoreactivity were initially selected from each section and then the EGFP channel was viewed, and the presence or absence of EGFP in each bouton was noted. In order to test for possible sampling bias, we measured the z-axis lengths of a sample of 60 boutons of each type (VGLUT2^+^/EGFP^+^/GRP^+^, VGLUT2^+^/EGFP^+^/GRP^−^ and VGLUT2^+^/EGFP^-^/GRP^+^), by selecting 10 of each type from each of the sections analysed.

Since we found evidence that the GRP antibody may cross-react with substance P (see below), we were concerned that the GRP immunoreactivity that we detected in EGFP^+^ axons could have resulted from binding of the antibody to a tachykinin peptide (substance P, neurokinin A or neurokinin B). These peptides, which share the same C-terminal amino acid sequence, are derived from two different genes, which code for preprotachykinin A (PPTA, which gives rise to both substance P and neurokinin A, NKA) and preprotachykinin B (PPTB, which generates neurokinin B, NKB). PPTA and PPTB are expressed by largely non-overlapping populations of excitatory neurons in the superficial dorsal horn [35–38], while PPTA (but not PPTB) is expressed by many nociceptive primary afferents [31, 32, 39, 40]. We therefore reacted transverse sections from the L2 segment with chicken anti-EGFP, rabbit anti-VGLUT2, rat anti-substance P and guinea pig anti-PPTB. One section from each animal was scanned through the 40× lens to generate a z-stack of ~20 optical sections at 0.5 μm z-spacing. These were opened in Neurolucida for confocal, with only the EGFP and VGLUT2 channels visible, and 100 EGFP^+^/VGLUT2^+^ boutons were selected from each animal. The channels corresponding to substance P and PPTB were then viewed, and the presence or absence of these peptides was noted for each of the selected boutons.

Because some excitatory interneurons in the superficial dorsal horn express protein kinase Cγ (PKCγ) [41], we tested whether this population included any of the GRP-EGFP cells. Transverse sections of L4 were reacted with chicken anti-EGFP, guinea pig anti-PKCγ and mouse monoclonal antibody NeuN. Three sections from each mouse were scanned through the 40× lens to produce z-series (20 sections, 1 μm z-separation) that covered the entire width of the superficial dorsal horn. The EGFP and NeuN channels were initially viewed with Neurolucida, and GRP-EGFP cells were selected as described above. The PKCγ channel was then examined, and the proportion of GRP-EGFP cells that were PKCγ^+^ was determined.

Finally, since populations of interneurons in the superficial dorsal horn have been classified based on morphology [24, 34, 42–48], we carried out a preliminary survey of the morphology of the GRP-EGFP cells by reacting sagittal sections through the L3 segment of both animals with chicken anti-EGFP. Three sections from each animal were scanned with the 40× lens through the full thickness of the section to produce z-stacks of 0.5 μm z-separation. These were examined in the original z-series, and also as maximum intensity projections.

#### Characterisation of antibodies

The GRP antibody was raised against the C-terminal sequence [14–27] of human GRP (MYPRGNHWAVGHLM) conjugated to bovine serum albumin (BSA) with glutaraldehyde. This sequence is identical to that of the corresponding region of mouse GRP, apart from a single amino acid (N for S at position 19). The antibody against CGRP was directed against synthetic rat α CGRP conjugated to BSA, and is blocked by pre-incubation with the incubating peptide at 10^−7^ M (manufacturer’s specification). The guinea pig, rabbit and goat antibodies against VGLUT2 were raised against peptides corresponding to amino acids 565–582 of rat VGLUT2 (guinea pig antibody) and amino acids 510–582 (rabbit antibody) or 550–582 (goat antibody) of mouse VGLUT2, respectively. The guinea pig antibody stains identical structures to the rabbit antibody [23], and both goat and rabbit antibodies detect a single protein band of the appropriate molecular weight (60 kDa) [49, 50]. The somatostatin antibody was raised against residues 1–12 of somatostatin-24, and detects identical structures to those labelled with a different somatostatin antibody, raised in rabbit [51]. The monoclonal substance P antibody detects the C-terminal 5–8 amino acids of the peptide [52], and does not appear to recognise NKB when used at concentrations sufficient to reveal substance P [35, 38]. The PPTB antibody was raised against a synthetic 40 amino acid peptide corresponding to residues 50–79 of PPTB, which was conjugated to human serum albumen with glutaraldehyde [53]. Immunostaining with this antibody is blocked by preincubation with the immunising peptide, but not by preincubation with substance P, NKA or NKB [53]. This antibody was used as it allows the identification of cells that express PPTB, and therefore presumably release NKB. The EGFP antibody was raised against recombinant full-length EGFP, while the antibody against Pax2 was raised against amino acids 188–385 of the mouse protein. The guinea pig antibody against PKCγ was raised against the C terminal 14 amino acids of the mouse protein and recognises a single band of the appropriate molecular weight on Western blots of brain homogenates from wild-type, but not PKCγ^−/−^ mice [54]. The mouse monoclonal antibody NeuN was raised against cell nuclei extracted from mouse brain [55] and apparently labels all neurons but no glial cells in the rat spinal dorsal horn [56].

#### Statistics

Differences in the z-axis lengths of different neurochemical types of axonal bouton were compared by using either a *t*-test or one-way ANOVA.

## Results

### Immunostaining with GRP antibody in the dorsal horn

**Figure 1:**
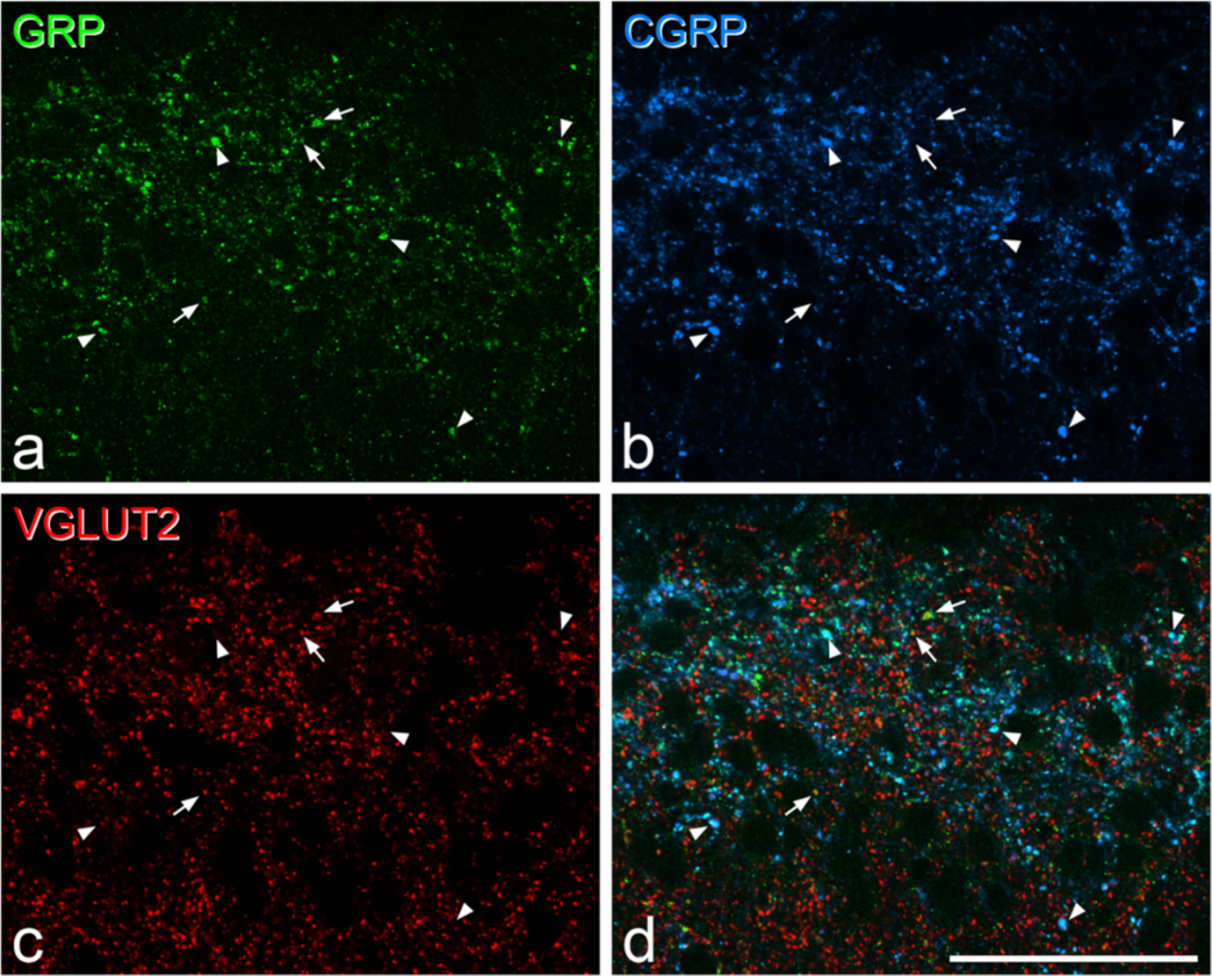
**The relationship between GRP, CGRP and VGLUT2 in laminae I-II. a-c**, Immunostaining with antibodies against GRP (green), CGRP (blue) and VGLUT2 (red) in a projection of two confocal sections (0.5 μm z-separation). **d** shows a merged image. At this magnification, the distribution of GRP immunoreactivity resembles that of CGRP, and many profiles are double-labelled (some shown with arrowheads). However, there are also GRP-immunoreactive profiles that lack CGRP and are strongly stained with the VGLUT2 antibody, and three of these are indicated with arrows. Scale bar = 50 μm.

Immunostaining with the GRP antibody resembled that reported in previous studies with antibodies against GRP or bombesin [5, 8, 57]. There was a dense band of staining in the superficial part of the dorsal horn, mainly in laminae I-IIo, and this was restricted to structures that resembled axons (Figure 1). No GRP immunoreactivity was seen in cell bodies.

**Figure 2:**
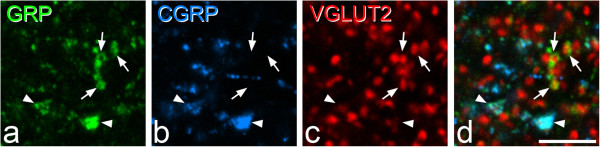
**High magnification view of GRP, CGRP and VGLUT2 in the superficial dorsal horn. a-c**, Immunostaining with antibodies against GRP (green), CGRP (blue) and VGLUT2 (red) in a single optical section. **d** shows a merged image. Several GRP-immunoreactive profiles are visible in this field. Two of these are CGRP^+^ and are stained very weakly with the VGLUT2 antibody (arrowheads), while several lack CGRP but show strong VGLUT2 immunoreactivity (three indicated with arrows). Scale bar = 10 μm.

In sections reacted with antibodies against GRP, CGRP and VGLUT2, we found numerous boutons that were immunoreactive for both GRP and CGRP, and these typically showed strong GRP immunoreactivity. However, we also identified many GRP-immunoreactive profiles that lacked CGRP, and these were frequently immunoreactive with the VGLUT2 antibody (Figures 1 and 2). Quantitative analysis revealed that GRP immunoreactivity was present in 70.1% (range 66.7-75.7%, n = 3 mice) of CGRP^+^ boutons in laminae I-IIo. As reported previously [23], we found that VGLUT2 immunoreactivity in CGRP^+^ boutons was either very weak or undectable, even though all primary afferents are thought to be glutamatergic [58, 59]. GRP immunoreactivity was seen in 30.2% (27.3-32%) of the VGLUT2^+^/CGRP^−^ profiles in laminae I-IIo, and the intensity of GRP labelling in these was often lower than that seen in the CGRP^+^ profiles. The mean z-axis length of GRP^+^/VGLUT2^+^ boutons was 1.53 ± 0.51 (n = 276 boutons), while that of the GRP^-^/VGLUT2^+^ boutons was 1.48 ± 0.47 (n = 624 boutons). These were not significantly different (p = 0.19, *t*-test), suggesting that our estimate was unlikely to be affected by a sampling bias towards larger boutons.

**Figure 3:**
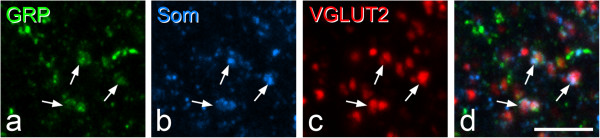
**Co-localisation of somatostatin and GRP in axons with strong VGLUT2 immunoreactivity. a-c**, Immunostaining with antibodies against GRP (green), somatostatin (Som, blue) and VGLUT2 (red) in a projection of 5 confocal sections (0.3 μm z-separation). **d** shows a merged image. Several profiles showing all three types of immunoreactivity are visible in this field, and 3 of these are indicated with arrows. Scale bar = 5 μm.

In the sections reacted for GRP, VGLUT2 and somatostatin, 70.2% (68-72%) of the GRP^+^/VGLUT2^+^ boutons in laminae I-IIo (axons of presumed GRP^+^ local neurons, see above) were somatostatin-immunoreactive (Figure 3). The sizes of boutons with and without somatostatin were 1.93 ± 0.45 μm and 1.84 ± 0.51 μm, and again, these were not significantly different (p = 0.06).

**Figure 4:**
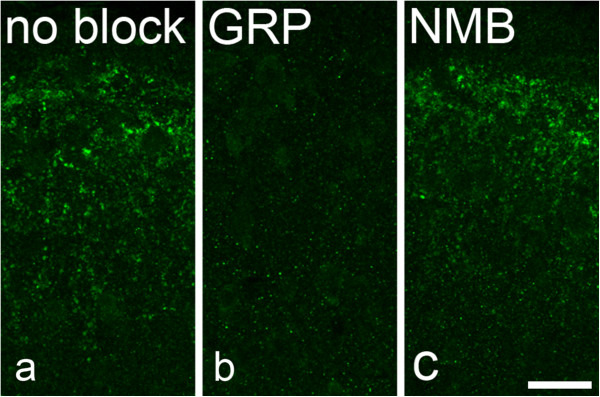
**The effect of pre-incubating the GRP antibody with GRP and NMB. a**, Immunostaining with the GRP antibody in the superficial dorsal horn from a transverse section. **b**, An equivalent field from a section in which the GRP antibody had been preincubated with GRP at 10^−7^ M overnight. **c**, Staining with antibody that had been preincubated with NMB at 10^−5^ M overnight. Note that preincubation with GRP completely blocked specific staining, whereas the staining was not affected by NMB. Scale bar = 20 μm.

**Figure 5:**
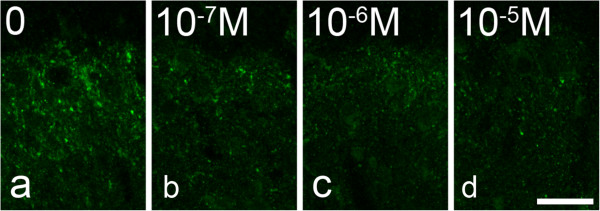
**The effect of pre-incubating the GRP antibody with different concentrations of substance P. a**, Immunostaining with the GRP antibody in lamina I-IIo from a transverse section. **b-d**, Equivalent fields from sections incubated in GRP antibody that had been preincubated overnight with different concentrations of substance P. Note that staining is diminished by preincubation with 10^−7^ M substance P, and largely absent at 10^−6^ and 10^−5^ M. Scale bar = 20 μm.

To test for the possibility that GRP immunoreactivity in peptidergic primary afferents represented a cross-reaction with either NMB [8] or a tachykinin peptide [9], we pre-incubated the anti-GRP antibody with either GRP, NMB or substance P, as described above. Pre-incubating the anti-GRP antibody with GRP completely blocked immunostaining, even at the lowest concentration tested (10^−7^ M), whereas immunostaining was not affected by pre-incubation with NMB, even at the highest concentration tested (10^−5^ M, Figure 4). Pre-incubation with substance P at 10^−5^ M largely blocked GRP immunostaining, and this was also reduced at 10^−6^ and 10^−7^ M (Figure 5).

### GRP-EGFP cells

**Figure 6:**
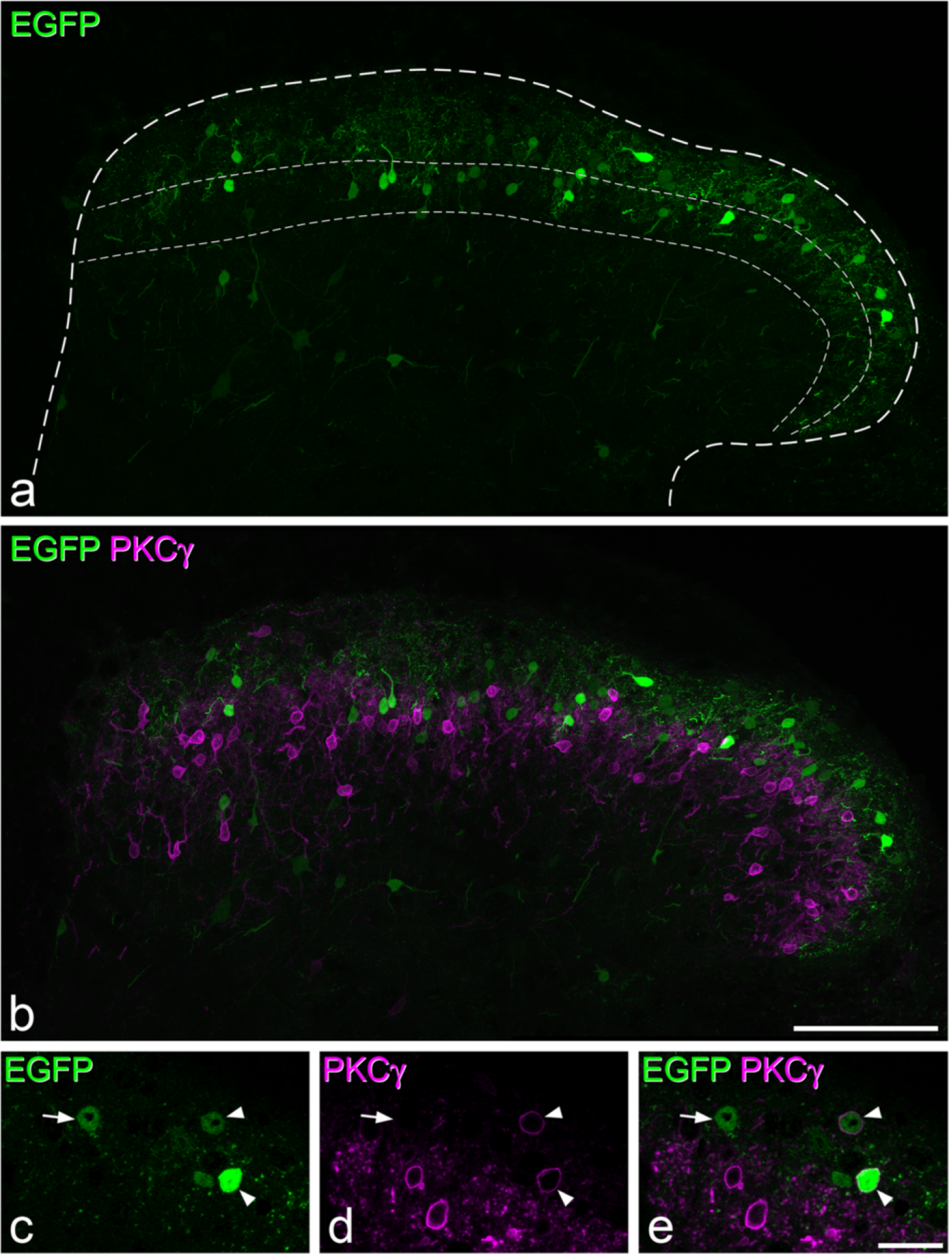
**Distribution of EGFP cells in the GRP-EGFP mouse. a**, Immunostaining for EGFP (green) in a transverse section shows scattered cell bodies throughout laminae I-II (projection of 20 optical sections at 1 μm z-spacing). The outer dashed line represents the outline of the dorsal horn, while the region between inner two lines corresponds to the inner half of lamina II, as defined by the plexus of PKCγ^+^ dendrites. **b** shows the same field scanned to reveal EGFP and PKCγ (magenta). Note that these are expressed in largely non-overlapping neuronal populations. **c-e**, Immunostaining with antibodies against EGFP (green) and PKCγ (magenta) showing the inner part of lamina II in a single optical section. Two EGFP^+^ cells that show weak PKCγ-immunoreactivity are indicated with arrowheads, and an EGFP^+^ cell that lacks PKCγ is marked with an arrow. Scale bars = 100 μm **(a,b)** and 20 μm **(c-e)**.

**Figure 7:**
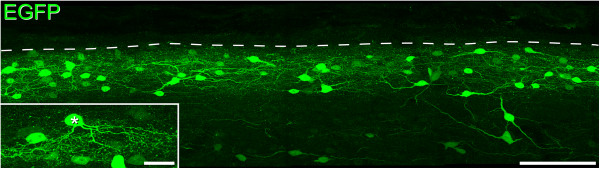
**Appearance of EGFP cells in the GRP-EGFP mouse in sagittal section.** Immunostaining for EGFP in a sagittal section (projected image through full thickness of a 60 μm section) reveals the dense band of GRP-EGFP cells that occupy laminae I-II, with scattered cells below this level. Note that the cells are morphologically diverse, with no obvious characteristic orientation of their proximal dendrites. Inset: a projected image (26 optical sections at 0.5 μm z-spacing) from a different section shows an EGFP-labelled cell with ventrally directed dendrites, which could be classified as a vertical cell. Scale bar = 100 μm for main image, 20 μm for inset.

As described by Mishra and Hoon [10], numerous EGFP^+^ cells were present in the dorsal horn of the GRP-EGFP mice, and these were concentrated in laminae I-II, with scattered cells present in the deeper laminae (Figures 6 and 7). The laminar distribution could be seen best in the transverse sections that had been reacted for PKCγ (Figure 6), as the plexus of PKCγ-immunoreactive dendrites can be used to define the inner half of lamina II [60]. As reported previously [10], we did not see EGFP-positive cells in the dorsal root ganglia of these mice (data not shown).

**Figure 8:**
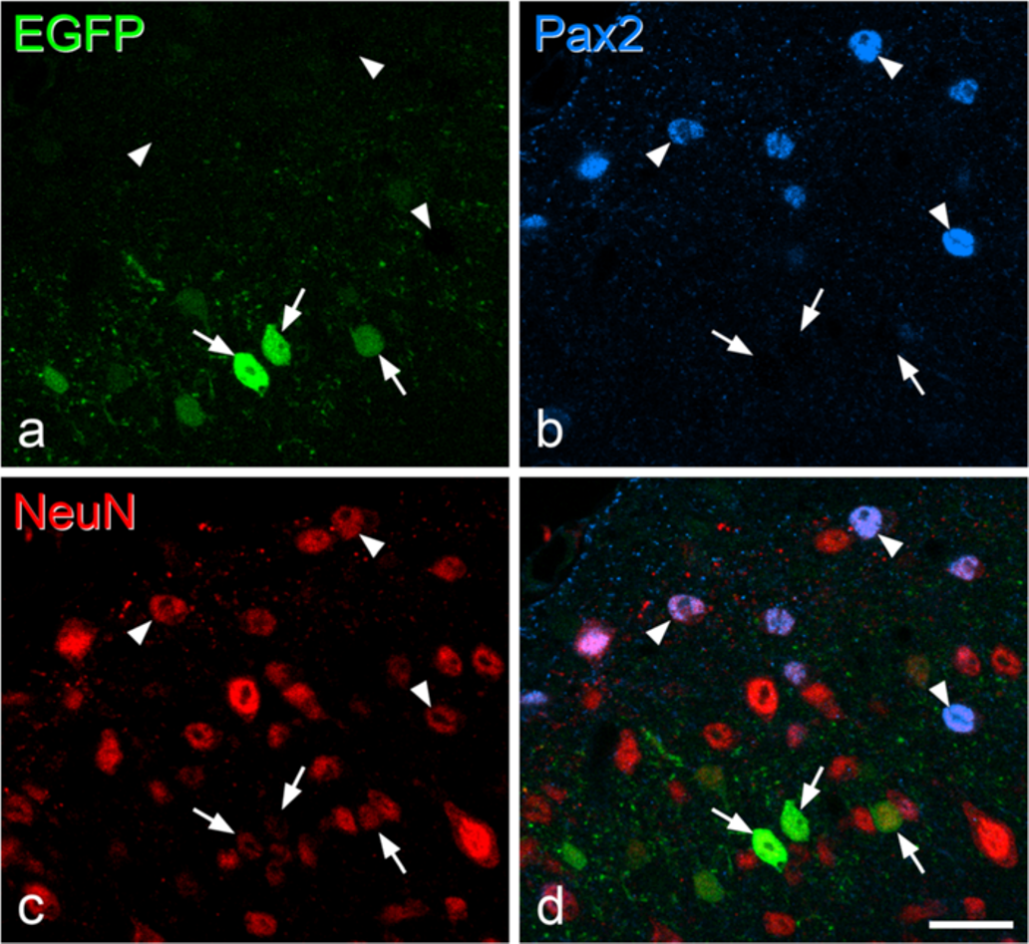
**Lack of Pax2 in EGFP cells in the GRP-EGFP mouse. a-c**, Immunostaining with antibodies against EGFP (green), Pax2 (blue) and NeuN (red) in a single optical section. **d** shows a merged image. Several EGFP^+^ cells, all of which are Pax2^−^ are visible, and 3 of these are marked with arrows. Note the very weak NeuN staining in these cells, compared to that seen in other nearby neurons, including those that are Pax2^+^, some of which are indicated with arrowheads. Scale bar = 50 μm.

Altogether, 180 EGFP cells were identified in the superficial dorsal horn in sections reacted for Pax2 and NeuN (125 and 55 cells in the 2 mice), and none of these was Pax2-immunoreactive (Figure 8), indicating that they are unlikely to be inhibitory interneurons. Interestingly, the staining for NeuN in the GRP-EGFP cells was consistently weak, compared to that seen in many EGFP-negative neurons. The consistently low level of NeuN probably reflects the fact that the GRP cells originate form a distinct developmental population, and has also been observed for γ-motoneurons in the ventral horn [61].

**Figure 9:**
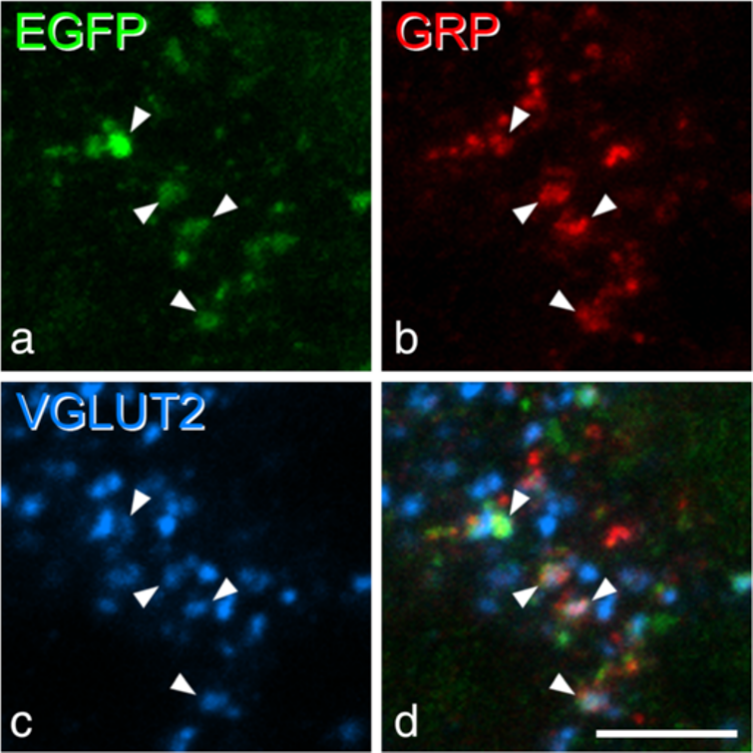
**GRP expression in EGFP cells in the GRP-EGFP mouse. a-c**, Immunostaining with antibodies against EGFP (green), GRP (red) and VGLUT2 (blue) in a projected confocal image (4 optical sections at 0.3 μm z-spacing). **d** shows a merged image. Four profiles showing all three types of immunoreactivity are marked with arrowheads. Scale bar = 5 μm.

**Figure 10:**
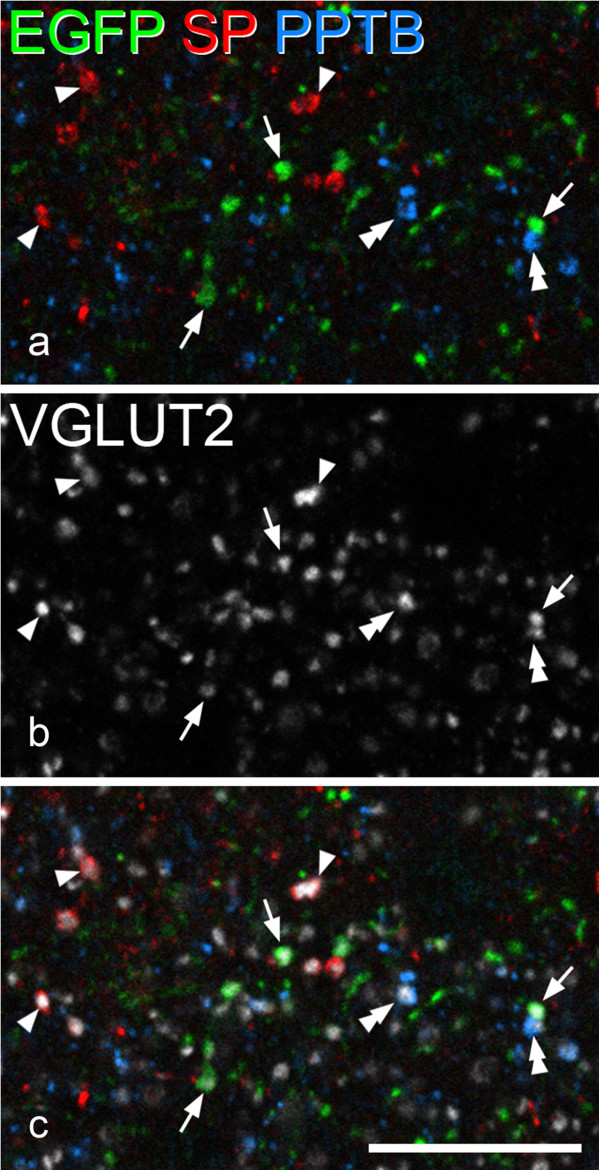
**Lack of co-localisation of EGFP and either substance P or PPTB in glutamatergic boutons in the GRP-EGFP mouse. a** Immunostaining with antibodies against EGFP (green), substance P (SP, red) and PPTB (blue) in a single optical section through lamina II from the L2 segment of one of the GRP-EGFP mice. **b** shows the same field scanned to reveal VGLUT2 (grey). **c** a merged image. Several VGLUT2-immunoreactive profiles that also stain for EGFP are visible, and 3 of these are indicated with arrows. These are not stained for either substance P or PPTB. Other VGLUT2 boutons that are stained with the substance P or PPTB antibodies are also present in this field, and some of these are marked with arrowheads or double arrowheads, respectively. Scale bar = 10 μm.

In the sections reacted to reveal EGFP, GRP, VGLUT2 and VGAT, numerous boutons with VGLUT2 or VGAT were present, and these formed non-overlapping populations. As described above, we found that GRP and VGLUT2 were frequently co-localised in boutons, whereas co-localisation of GRP and VGAT was extremely rare (only 6 examples of GRP^+^/VGAT^+^ profiles were seen throughout the full depth of the 6 z-stacks analysed in this part of the study, and we estimate that this corresponds to around 0.1% of the GRP boutons). This supports the suggestion that GRP expression by superficial dorsal horn neurons is largely restricted to those that are excitatory [11]. EGFP was never co-localised with VGAT in these sections, providing further evidence that the EGFP cells were not inhibitory interneurons. In contrast, EGFP and VGLUT2 were frequently colocalised (Figures 9 and 10). GRP-immunoreactivity was detected in 72.3% (69.3% and 75.3% in the two mice) of the profiles that contained both EGFP and VGLUT2. Conversely, EGFP was seen in 41.7% (46%, 37.3%) of profiles that were immunoreactive for both VGLUT2 and GRP. The mean z-axis lengths of EGFP^+^/GRP^+^, EGFP^+^/GRP^−^ and EGFP^−^/GRP^+^ boutons in this sample were 1.88 μm ± 0.51 μm, 1.83 μm ± 0.44 μm and 1.94 μm ± 0.47 μm. These did not differ significantly (one-way ANOVA, p = 0.49), indicating that our results were not likely to have been affected by a sampling bias due to size differences between the different populations.

In sections reacted to reveal EGFP, VGLUT2, substance P and PPTB, we found virtually no expression of either substance P or PPTB among the EGFP^+^/VGLUT2^+^ boutons (Figure 10). Only 1 of the 200 selected boutons was also immunoreactive for PPTB, and none for substance P. Although many VGLUT2 boutons showed either substance P or PPTB immunoreactivity, we found very few that were stained for both peptides (as reported previously for the rat [35, 38]). Since NKA is apparently restricted to neurons that also express substance P [62], the lack of either SP or PPTB immunoreactivity in the boutons of the GRP-EGFP cells indicates that the GRP immunoreactivity that we observed in them is not due to cross-reactivity with a tachykinin peptide, and is therefore likely to be genuine.

In the sections reacted for PKCγ, there was some overlap in the distribution of EGFP^+^ and PKCγ^+^ cells in lamina IIi, although many EGFP^+^ cells were located dorsal to the main band of PKCγ^+^ cells (Figure 6). In these sections, 112 EGFP cells were selected in one animal and 73 in the other. The great majority of the EGFP cells were not PKCγ-immunoreactive, although a few (15.2% and 9.6%, respectively) showed weak PKCγ immunoreactivity (Figure 6c-e).

Numerous EGFP-labelled cells were seen in the sagittal sections, and these varied considerably in the strength of staining (Figure 7). For strongly labelled cells it was possible to follow dendritic trees for some distance, and dendritic spines could easily be identified. The EGFP-labelled cells were morphologically heterogeneous, and in most cases dendrites were orientated along the rostrocaudal axis or travelled obliquely in both rostrocaudal and dorsoventral axes. A small number of EGFP-labelled neurons resembled vertical cells, and an example is shown in Figure 7 (inset).

## Discussion

The main findings of this study are: (1) that immunostaining with a GRP antibody is present in both primary afferents and non-primary glutamatergic axons in the superficial dorsal horn, (2) that immunostaining is partially blocked by pre-incubation with substance P, but not with NMB, (3) that EGFP-labelled cells in the GRP-EGFP mouse line are Pax2-negative, with most lacking PKCγ, and (4) that many of the axons derived from EGFP-labelled cells in this line express detectable levels of GRP-immunoreactivity, but are not immunoreactive with antibodies against substance P or PPTB.

### Sources of GRP-immunoreactive axons in the superficial dorsal horn

Our finding that GRP immunoreactivity could be detected in the majority of CGRP^+^ axons in laminae I-IIo is consistent with several previous reports that other antibodies raised against GRP or the related peptide bombesin stain cell bodies of peptidergic primary afferents in the dorsal root ganglion [5, 57, 63–66], and that there is significant depletion of immunostaining in the dorsal horn following dorsal rhizotomy [8, 57]. However, although GRP mRNA has been identified in dorsal root ganglion cells with *in situ* hybridisation and the polymerase chain reaction by the Chen laboratory [5, 20], studies from two other groups have failed to detect significant levels of the mRNA in dorsal root ganglia using either these methods or quantitative RNA sequencing [8–10]. The question of whether GRP is actually expressed in primary afferents therefore remains controversial. Fleming et al. [8], noting the high level of expression of NMB in dorsal root ganglia and the close sequence similarity between NMB and GRP, suggested that much of the GRP immunoreactivity identified in primary afferents could result from cross-reaction of the GRP antibodies with NMB. However, we found that relatively high concentrations of NMB (10^−5^ M) were unable to block immunostaining with our anti-GRP antibody, even though this was completely blocked by GRP at 100 times this dilution (10^−7^ M). It is therefore unlikely that the primary afferent staining that we saw with anti-GRP represents a cross-reaction with NMB. Goswami et al. [9] suggested that GRP antibodies might cross-react with tachykinin peptides, such as substance P, which are highly expressed in primary afferents, and which show some similarity in their C-terminal amino acid sequence to GRP (−GLM in tachykinin peptides, compared to -GHLM in GRP and -GHFM in NMB). In addition, we had found in preliminary studies that including anti-substance P in the primary antibody mixture reduced the staining for GRP in these sections (MGM and AJT, unpublished data). We therefore tested the effects of pre-incubating with substance P on the staining seen with the GRP antibody, and found that this was suppressed. Although we cannot be certain that the GRP antibodies would bind to substance P in fixed tissue, this observation raises the possibility that the GRP-like immunoreactivity seen in peptidergic primary afferents in this study may represent a cross-reaction with substance P or the closely related peptide NKA. It is also possible that previous reports of primary afferent labelling with different GRP antibodies [5, 57, 63–66] may have resulted, at least in part, from cross-reaction with tachykinin peptides. This could be tested in future studies by analysis of tissue from mice in which the gene for PPTA has been knocked out [67].

GRP-immunoreactivity was also detected in boutons that were VGLUT2^+^ but lacked CGRP, and most of these are likely to originate from local excitatory interneurons [23, 24]. Consistent with this suggestion, we found that somatostatin, which is expressed by many excitatory interneurons in the superficial dorsal horn [25], was frequently co-localised with GRP in VGLUT2^+^ axons. It is likely that at least some of this represents genuine GRP, as it was sometimes co-localised with EGFP in axonal boutons in the GRP-EGFP mouse, and these cells did not express either of the two preprotachykinins.

Since only ~40% of the GRP^+^/VGLUT2^+^ boutons in this mouse line were immunoreactive for EGFP, it is possible that the staining in other non-primary (i.e. VGLUT2^+^/EGFP^−^) axonal boutons resulted from cross-reaction of the GRP antibody with tachykinin peptides, which are expressed by many excitatory neurons in this region [35–37]. However, the failure to detect EGFP in these boutons may alternatively have resulted from weak expression (or lack of expression) of EGFP in some GRP-containing cells in this BAC transgenic line.

### GRP-expression by excitatory interneurons

Several studies have reported that large numbers of cells with GRP mRNA are present in the superficial dorsal horn [8, 10–15]. It has also been shown that these are likely to be excitatory neurons, since their survival is dependent on expression of transcription factors that are involved in determining a glutamatergic phenotype [11, 12, 15]. In addition, it has been reported that most of the GRP-expressing cells are lost in mice lacking the testicular orphan nuclear receptor (TR4), in which there is preferential depletion of excitatory interneurons [14]. However, Liu et al. [20] recently claimed that cells with GRP mRNA were restricted to lamina I and state that “to date, little evidence exists to support that Grp mRNA in lamina I of the spinal cord is in fact translated into GRP protein”. This statement was apparently based on the failure of immunocytochemical studies to demonstrate GRP-immunoreactivity within cell bodies in the superficial dorsal horn. However, although some of the neuropeptides expressed by neurons in this region can be identified in their cell bodies with immunocytochemistry [25, 68–70], others, such as substance P, NKB, dynorphin or the enkephalins are either not detected, or else are seen in very few cells. The failure to detect somatic staining for these peptides results from rapid transport of the peptide out of the cell body, and for this reason colchicine was administered in several early studies to prevent axoplasmic transport, therefore elevating the levels of neuropeptides in cell bodies [71, 72].

Our finding that ~70% of the axon terminals that contained EGFP in the GRP-EGFP mouse were GRP-immunoreactive (and that they lacked tachykinins, with which the GRP antibody can cross-react) strongly suggests that these cells are indeed translating the mRNA into GRP. The lack of immunoreactivity in the other ~30% of EGFP^+^/VGLUT2^+^ boutons may reflect variable levels of GRP expression, with some axons containing amounts below the detection threshold of the GRP antibody. Alternatively it could result from ectopic expression of EGFP in this line. However, even if there is ectopic EGFP expression, it is evidently not random, as it seems to be restricted to excitatory (Pax2^−^) cells.

Excitatory interneurons account for ~70% of the neurons in the superficial dorsal horn [73], and are thought to include several distinct subpopulations that are likely to have differing roles in the processing of sensory information [14, 59, 74–76]. Several attempts have been made to define these functional populations, using morphological, electrophysiological, neurochemical and/or developmental approaches [12, 24, 34, 44–47, 77–79]. Although the neurons in this region are morphologically diverse, certain distinctive classes have been identified among the excitatory cells in lamina II. These include vertical cells, which have dendrites that project ventrally from the soma and an axon that often enters lamina I where it may be presynaptic to projection neurons, and radial cells, which are small and have short radiating dendrites [24, 34, 44–47, 80]. A third morphological group (central cells), has also been described [34, 44–46]. These have relatively short dendritic trees orientated along the rostrocaudal axis. However, central cells are a more diverse set that includes both excitatory and inhibitory interneurons [24]. A limitation of this scheme is that all morphological studies have found a significant fraction of lamina II interneurons (typically ~25%) that cannot be be assigned to any of these morphological classes [24, 34, 44–46]. Based on the appearance of GRP-EGFP cells in sagittal sections, most did not appear to be vertical or radial cells, and so they are likely to belong to central or unclassified populations.

A neurochemical approach has proved useful in defining functional subsets among the inhibitory interneurons in the superficial dorsal horn. For example, we have identified four largely non-overlapping populations, those that express neuropeptide Y (NPY), galanin, neuronal nitric oxide synthase (nNOS) and parvalbumin, and found that these differ in their responses to noxious stimulation [27]. Many of the cells that contained NPY or galanin were activated by noxious heat, as well as by subcutaneous injection of formalin or capsaicin. In contrast, the nNOS-containing cells responded to noxious heat and formalin injection, but seldom to capsaicin, while the parvalbumin cells were not activated by any of these stimuli. In addition, it has been shown that the parvalbumin-expressing cells give rise to axoaxonic synapses on myelinated low-threshold mechanoreceptive afferents [81], while the nNOS and/or galanin populations have been implicated in the suppression of itch by counterstimuli [33].

Several neurochemical markers are expressed by subsets of excitatory neurons, and it would be of value if we could also define non-overlapping populations among these cells. It had already been shown that there is little co-localisation of PPTB and substance P in axons in laminae I-II [35, 38], and taken together with the results of this study, it therefore appears that different sets of excitatory neurons express PPTA, PPTB and GRP. Neurotensin is also present in some excitatory neurons in laminae IIi-III [23, 82]. These are known to be different from those that express PPTB [35], and we have found little or no co-localisation of neurotensin and substance P (MGM and AJT, unpublished data). Since most of the neurotensin-containing cells also express PKCγ [41], it is likely that they are largely separate from the cells with GRP, as the EGFP^+^ cells seen in this study were generally PKCγ-negative. This suggests that there may be four distinct neuropeptide-expressing populations among the excitatory neurons in laminae I-III: those with PPTA, PPTB, GRP and neurotensin. Since the axonal boutons of these cells can be recognised by co-localisation of the corresponding peptide with VGLUT2, it should be possible to use immunocytochemistry to investigate the postsynaptic targets for each population. Nothing is apparently known yet about the responses of the GRP-expressing interneurons to peripheral stimuli, but as GRP and the GRPR have been implicated in itch [5, 6], it will be important to determine whether the GRP-expressing neurons in laminae I-II are activated by pruritic stimuli.

## Conclusions

The results of the present study indicate that GRP is expressed by at least some of the neurons that contain EGFP in the GENSAT mouse line Tg(GRP-EGFP). They also show that these cells are excitatory interneurons, and are largely different from those that express PPTA, PPTB or PKCγ. In addition, they raise the possibility that some of the GRP immunoreactivity seen in this and previous studies may have resulted from a cross-reaction with substance P and/or NKA.

## Abbreviations

BSA: Bovine serum albumin
CGRP: Calcitonin gene-related peptide
DRG: Dorsal root ganglion
EGFP: Enhanced green fluorescent protein
GRP: Gastrin-releasing peptide
GRPR: Gastrin-releasing peptide receptor
NKA: Neurokinin A
NKB: Neurokinin B
NMB: Neuromedin B
nNOS: Neuronal nitric oxide synthase
NPY: Neuropeptide Y
PKCγ: Protein kinase Cγ
PPTA: Preprotachykinin A
PPTB: Preprotachykinin B
VGAT: Vesicular GABA transporter
VGLUT2: Vesicular glutamate transporter 2.
